# Development of risk maps to minimize uranium exposures in the Navajo Churchrock mining district

**DOI:** 10.1186/1476-069X-8-29

**Published:** 2009-07-09

**Authors:** Jamie L deLemos, Doug Brugge, Miranda Cajero, Mallery Downs, John L Durant, Christine M George, Sarah Henio-Adeky, Teddy Nez, Thomas Manning, Tommy Rock, Bess Seschillie, Chris Shuey, Johnnye Lewis

**Affiliations:** 1 Tufts University School of Engineering, Department of Civil and Environmental Engineering, 200 College Ave Anderson Hall, Medford, MA, 02155, USA; 2 Tufts Community Research Center, Department of Public Health and Family Medicine, 136 Harrison Ave Boston, MA, 02138, USA; 3 Community Environmental Health Program, College of Pharmacy, MSC09 5360, 1 University of New Mexico Albuquerque, NM, 87131-0001, USA; 4 50 Haven Ave B924, NY, 10032, USA; 5 Southwest Research and Information Center, Uranium Impact Assessment Program, 105 Stanford SE PO Box 4524, Albuquerque, NM, 87196, USA; 6 Eastern Navajo Health Board, PO Box 1938, Crownpoint, NM, 87313, USA; 7 College of Pharmacy, University of New Mexico, 2505 Marble NE, Albuquerque, 87131-0001, New Mexico, USA

## Abstract

**Background:**

Decades of improper disposal of uranium-mining wastes on the Navajo Nation has resulted in adverse human and ecological health impacts as well as socio-cultural problems. As the Navajo people become increasingly aware of the contamination problems, there is a need to develop a risk-communication strategy to properly inform tribal members of the extent and severity of the health risks. To be most effective, this strategy needs to blend accepted risk-communication techniques with Navajo perspectives such that the strategy can be used at the community level to inform culturally- and toxicologically-relevant decisions about land and water use as well as mine-waste remediation.

**Objective:**

The objective of this study was to develop GIS-based thematic maps as communication tools to clearly identify high risk exposure areas and offer alternatives to minimize public and ecological health impacts.

**Methods:**

Thematic maps were produced that incorporated data derived from environmental sampling and public health surveys. The maps show the location and quality of unregulated water resources and identify regulated water sources that could be used as alternatives. In addition, the maps show the location of contaminated soil and sediment areas in which disturbance of surface deposits should be avoided. Preliminary feedback was collected from an informal Navajo working group to assess the clarity and efficacy of this proposed communication method.

**Results:**

The working group found the maps to be both clear and effective, and made suggestions for improvements, such as the addition of more map features. The working group predicted that once the maps are presented to the public, water hauling and soil use behaviors will change, and dialogue with chapter officials will be initiated to accelerate further risk reduction efforts.

**Implications:**

Because risk communication is complicated by language barriers, lack of infrastructure, and historical mistrust of non-Navajo researchers, mapping provides an easily interpretable medium that can be objectively viewed by community members and decision makers to evaluate activities that affect toxicant exposures.

## Background

Risk assessments performed by the federal government, state agencies, and private contractors at hazardous waste disposal sites serve as a foundation for estimating human and ecologic health risks [1]. However, these assessments are often ineffective for native communities as they do not address cultural factors affecting contaminant exposure such as consumption of locally-raised livestock and native plants, and their use in traditional medicines and ceremonies [2]. In addition, commonly applied risk assessment methodologies often result in communication of risk management strategies that are not acceptable to native communities because the risk reduction practices are threatening to cultural practices and beliefs [3]. Furthermore, the lack of communication of study results to communities- particularly after concerns over exposure to toxic waste have been raised- has contributed to failed environmental health improvements in native communities [4].

Exposure scenarios appropriate for many native populations differ from those for the general U.S. population. Studies among other native groups including the Akwesasne Mohawk, Shoshone, Southern Paiute, Ouje-Bougoumou Cree, and Ojibwa have all employed alternative methods of exposure assessment and risk communication to address environmental hazards in these communities [5-8]. These studies not only highlight the benefits of collaborative methods in addressing environmental exposures, but also underscore common challenges facing different native groups particularly when risk reduction measures are recommended that would alter subsistence diets or cultural practices. Developing partnerships with communities that allow these assessments to be done in a way that respects cultural values and also ensures the data have the scientific validity to provide answers that address their health concerns can be a slow process. Efforts to broadly characterize exposures and risks from uranium in the context of known risk factors in Navajo communities have been developed based on interactions between researchers and community members that span more than 20 years. This exposure characterization is an initial step in research to determine the contribution of these exposures to the health status of the community members. The implementation of this research into health effects is in progress in an eight-year study that is only beginning to result in publications in the peer-reviewed literature [9]. The approach investigated here offers an additional alternative to integrated risk assessment which was developed out of the communication and frequent guidance among the university and community partners and research participants in twenty affected Navajo communities.

Churchrock chapter, a local government unit of the Navajo Nation in northwestern New Mexico, is familiar with historical research and risk assessment that has not been collaborative. For decades, residents of Churchrock have lived with a legacy of environmental contamination left behind from uranium mining and milling activities. After an earthen tailings dam failed in 1979, sending 1,100 tons of radioactive mill waste and 94 million gallons of acidic wastewater down the Puerco River, ecological studies were conducted to assess radionuclide contamination in water, sediment, vegetation and livestock [10-16]. Human health risks in these studies were estimated as lifetime cancer risk, and calculated using exposure assumptions developed for the U.S. as whole, not the Navajo specifically. These studies were based solely on radiological risk, ignoring the chemical toxicity of many of the contaminants of concern [17]. In addition, the studies made recommendations to the Navajo community to cease consumption of organ meat, stop drinking water from sources known to contain elevated uranium levels, and stay away from the river bank under dry or windy conditions. However, these were not realistic alternatives for Navajo residents who relied on local water sources that are not routinely monitored, consumed locally-grown animal meat, and herded livestock and gathered herbs in close proximity to the river.

**Figure 1:**
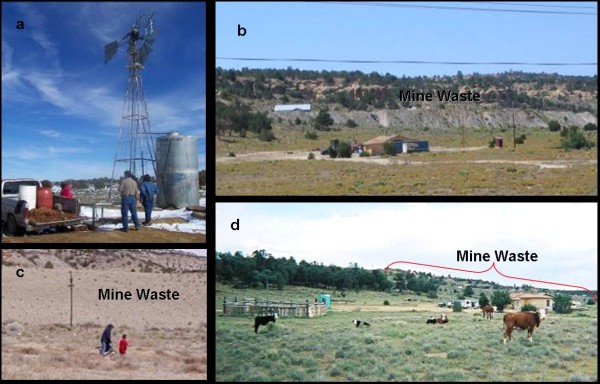
**Exposure conditions specific to most Navajo Communities**. a. Navajo family hauling water from a windmill.; b. new home under construction within 1200' of mine waste pile; c. children playing near partially reclaimed waste pile; d. cattle grazing on contaminated soil adjacent to mine waste. (Photo credit, J. deLemos, C. Shuey, and C. George)

Among the Navajo people, more than 50% of residents in the affected communities drink from unregulated water sources, and >80% of Navajo families haul drinking water, despite having municipal water supplied to their homes (Figure 1a) [18]. Some residences are within a few hundred feet of exposed uranium mining-waste piles, creating increased contact with contaminated sediments that have spread into residential areas (Figure 1b–c). Food chain contamination is also a concern, as mutton is a staple of the Navajo diet 19. and elevated levels of uranium have been detected in livestock and forage in Churchrock and other uranium-impacted areas 9; Figure 1d.

It is possible that exposure to uranium, a nephrotoxicant, may be contributing to an excess of kidney disease among the Navajo [20-22]. The age-adjusted prevalence of end-stage renal disease (ESRD) among the Navajo people is more than three times that of the general U.S. population [23]. Known risk factors for kidney disease, such as diabetes and hypertension, are also elevated among the Navajo, but alone cannot explain the severity, high prevalence, and early onset (often at ages less than 20) of chronic kidney disease [24,25]. These health problems, along with a lack of data describing the environmental distribution of uranium, prompted the Eastern Navajo Health Board, the University of New Mexico Community Environmental Health Program, and the Southwest Research and Information Center to jointly investigate environmental, health, socioeconomic, and cultural risk factors contributing to kidney disease. Two programs were initiated through this collaboration: the Churchrock Uranium Monitoring Project (CRUMP), and the Diné Network for Environmental Health (DiNEH) Project. Between 2003 and 2007, CRUMP collaborators focused on the chemical and radiological characterization of environmental media impacted by abandoned mines in residential areas in Churchrock and four adjoining Navajo chapters. Since 2004, the DiNEH Project has collected water and sediment data and conducted a health and water-use survey in these and 15 other chapters of the Eastern Agency of the Navajo Nation.

The collection of health and environmental data has been an important first step in identifying risk for persistent health problems in these communities. This effort has been aided significantly by the use of geographic information systems (GIS) for managing survey data, identifying areas of concern for environmental sampling, and mapping the locations of water resources in remote areas. Because GIS provides a means to understand spatial disease patterns in relation to the physical environment; the use of GIS in environmental epidemiology and risk assessment is rapidly increasing [26,27]. However, the role of thematic maps (produced with GIS) in risk communication, particularly in Native and rural communities, has not been widely studied. Few studies have employed GIS as a component of an integrated risk assessment 7; [28,29], and while these studies have highlighted the utility of thematic mapping for this purpose, they have not reported on community feedback regarding their potential efficacy as an intervention.

The need for clear, concise, and readily understood information is critical in these communities where the likelihood that the hazards will be addressed in a systematic and comprehensive manner is minimal in the near term. Soil and water in livestock wells are the primary sources of contamination. Residents spend substantial time out of doors engaged in various activities that bring them in contact with contaminated soils including walking or riding horses across the land, or herding sheep, cattle, and horses. The geographic area occupied by the Navajo Nation is roughly the size of West Virginia, and although soil contamination can be distributed across many areas of the reservation, it is likely to occur in pockets that allow residents to move via other routes if they know the location of contamination. With respect to water, only 30% of Navajos lack access to regulated water in their homes (compared to 0.6% of the general US population, and 12% of US tribal members as a whole), and therefore residents frequently haul water from numerous wells intended for livestock use only [30]. Knowing where contaminated soil areas and water sources exist can help the residents to make more informed choices and select alternative geographic areas for their activities that minimize the risk to their health.

The objectives of this investigation were to 1) utilize both environmental and water-use data collected through CRUMP and DiNEH projects to define land and water resources that may act as routes for toxicant exposure for the Churchrock community; and 2) integrate these data using GIS tools to provide thematic maps that communicate exposure risks in an easily interpretable manner. These maps are intended to support other ongoing outreach activities conducted by CRUMP and DiNEH initiatives (e.g., educational presentations at chapter houses, youth activities) and were created as a response to a community need for a more permanent, readily available and rapidly interpreted source of information in chapter houses where community members gather.

## Methods

### Study area

**Figure 2:**
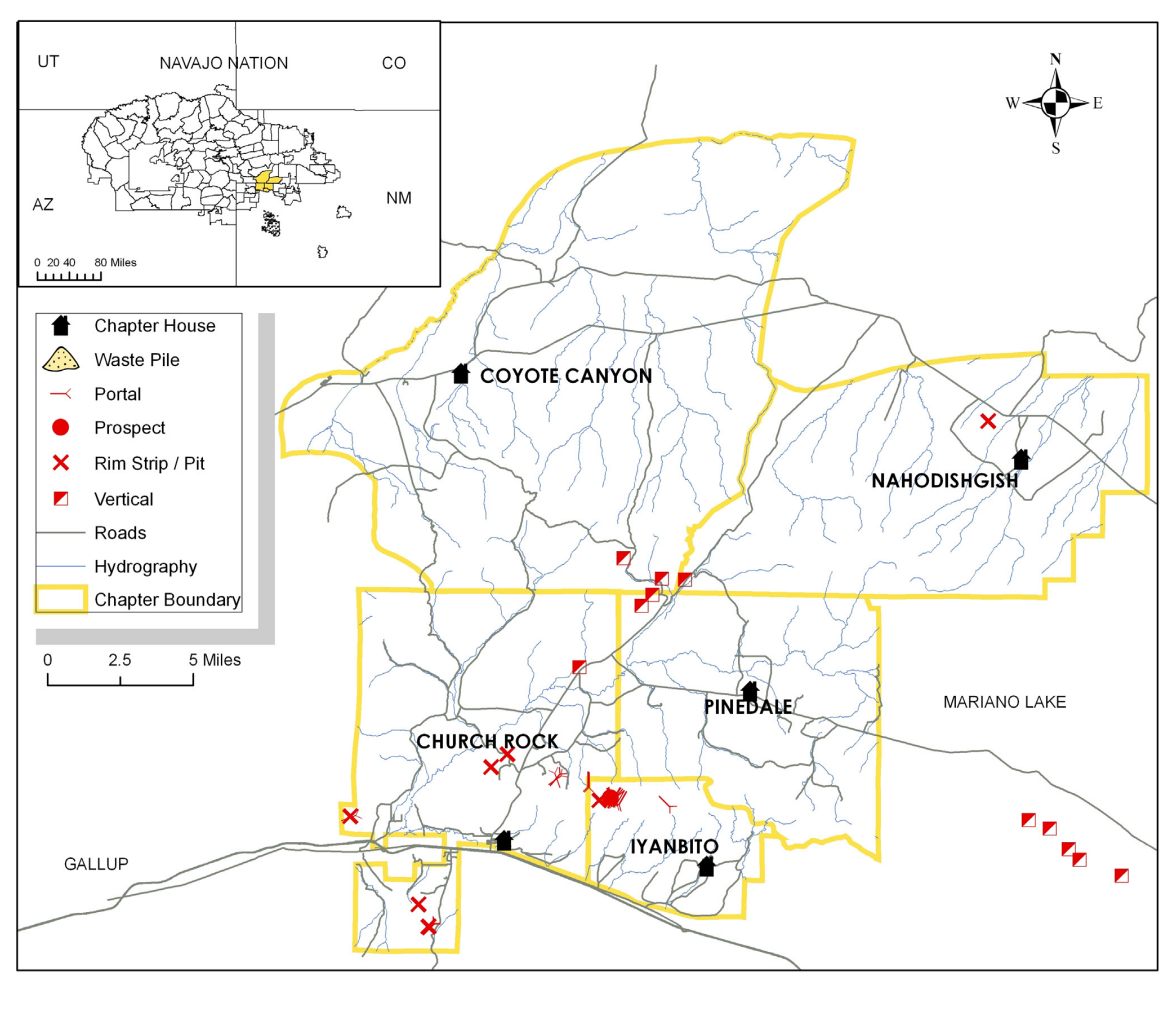
**Map of the study area including chapter boundaries, main roads, hydrography, location of chapter house, as well as mine features (portal- mine entrance; prospect-area of exploration, rim/pit- land stripped away to access ore; or vertical shaft-passage into a mine) and waste piles**.

The study area includes Churchrock and the adjacent Navajo communities Pinedale, Coyote Canyon, Iyanbito and Nahodishgish (New Mexico), all of which are chapters (political units) of the Eastern Agency of the Navajo Nation (Figure 2). These rural chapters span approximately 3,000 km^2^, and have low housing density and population (~ 6,000), few paved roads, and minimal infrastructure 2000 Census. Approximately 20 abandoned uranium mines (AUMs) are located in this area (Figure 2), with the largest former mines located on the boundary of Churchrock, Coyote Canyon, and Pinedale chapters [31,32]. These mine sites, many of which have not been reclaimed, produced more than 4 million tons of uranium ore between 1950 and the late 1980s [31]. Mine sites overlay common local and regional aquifers and drain to the intermittent north fork and main stem of the Puerco River. The regulation, assessment, and reclamation of abandoned mines has been complicated by the land status of the region, referred to as the "checkerboard" area for its mixture of tribal trust, fee, allotment, private, and federal land [33]. Because so many government entities are involved with enforcement of this region, there are substantive jurisdictional issues that arise when addressing responsibility for clean-up. Jurisdiction is further complicated by the history of uranium mining where, for much of the time all mining was conducted by private entities under contractual relationships with the US government to obtain ore for military purposes. Further complicating clean-up decisions, federal programs that prioritize clean-up projects, such as the Hazard Ranking System administered by the U.S. EPA, make it difficult for sites with low population densities to rank sufficiently high to be considered a priority for remedial action.

For the purposes of this investigation, the mapping area was not limited to the geographic region that falls within the designated Churchrock chapter boundary. Inclusion of surrounding chapters was necessary to encompass both the physiographic aspects controlling contaminant transport and exposure (e.g., drainage catchments), and the cultural and activity factors (e.g., horseback riding, herding sheep, and collecting water) that influence exposure. The area encompasses both lands free of and lands heavily burdened by mining impacts – equating to low and potentially high risk areas where people are likely to travel on a daily basis.

### DiNEH survey and participant demographics

The DiNEH project is a research collaborative partnership among 20 Navajo Chapters, the Eastern Navajo Health Board, the Southwest Research and Information Center, and the Indian Health Service Crownpoint Service Unit. The request for research was initiated by the Eastern Navajo Health Board in 2000, and they and other community members have been intimately involved in all aspects of the design of the multilevel research project that incorporates extensive characterization of exposure, health information, socioeconomic status, and family history as well as environmental sampling, medical record review, and clinical assessment. All staff are from the communities and form a regular interface with the community through regular participation in chapter meetings. The DiNEH survey was developed with Navajo community member participation, field-tested by bilingual Navajo community environmental health workers (CEHWs), and approved through Navajo Nation and University institutional review boards. The instrument has been revised iteratively based on feedback from staff. Questions were designed to ascertain uranium and other toxicant exposures and include inquiries on water and land use patterns, occupational histories, locations of residences and their proximity to contaminated sites, socioeconomic factors, cultural practices, health risk factors for kidney disease and medical status. Use of an oral survey instrument was the method employed by DiNEH project to collect quantitative data for a multi-level kidney risk model (results reported elsewhere), categorize past and current exposures, and provide a basis for developing exposure mitigation strategies.

Participants were recruited at water hauling locations, chapter meetings, public events, or by word of mouth, and were generally surveyed at the home, unless an alternative location was requested. Surveys were administered orally in Navajo, English, or a combination of both and were typically completed in one hour. The survey was implemented in 20 chapters of the Eastern Agency of the Navajo Nation. About 500 surveys were administered in the first phase of the project through December of 2007 800 more are in progress to be completed in the next two years. The mapping area we examine here represents a quarter of the overall DiNEH study area, and will be used as a prototype for map-based risk communication in the remaining chapters. To date, 151 residents have completed surveys within the mapping area. Summary demographics are reported in Table 1.

**Table 1:** Mapping area demographics

Total Participants (n)	151
% Male	42
% Female	58
Mean Age (± standard deviation)	58 ± 16
Language	
% Bilingual with family	58
% Bilingual at work	45
% Bilingual with friends	50
% Reporting annual household income <$15,000	63
% Reporting a high school diploma or higher level of education	35
Median fraction of life spent at current residence	0.7
Median # of minutes to access food and supplies	30
Median # of minutes to access medical services	25
Median # of minutes to water source	20
Median # of minutes to work	30

#### Self-reported activities influencing exposures

The goal of compiling survey data was to gauge which activities were most significant in influencing uranium exposures. While the survey takes into account occupational history, uranium mining and mill sites are no longer in operation and are not a current source of occupational exposure. While it is clear that occupational exposure to uranium and its daughter products can lead to lung disease and cancer and will be incorporated into the overall modeling effort, these occupational exposures are not occurring now and therefore are not included in the risk mapping communication strategy reported here [34]. Contact with contaminated material occurred historically and continues to be a present-day concern with the abundance of exposed waste in residential areas. Residential proximity to mine and mill sites also influences current exposures, as the median fraction of life at the current residence is 0.7, suggesting limited migration and increased probability for contact with contaminated material in the course of day-to-day activities. Water hauling information and use patterns may also affect exposure and be modified to minimize ingestion of contaminated water. Therefore risk mapping will focus on ways to adjust activities associated with water hauling and contact with contaminated sediments.

#### Water Hauling

Nearly 100% of participants report hauling water from at least 1 source, despite the fact that 66% of respondents are connected to Navajo Tribal Utility Authority (NTUA) water (water which is piped and regulated by the utility). Sixty-five percent report hauling from at least two sources, and 33% haul from 3 sources. Less than 10% of participants report hauling from 4 or more sources. Percentages reported from these 5 chapters may be skewed lower than percentages reported throughout the 20 chapter sample due to the close proximity of services in the City of Gallup, NM.

**Figure 3:**
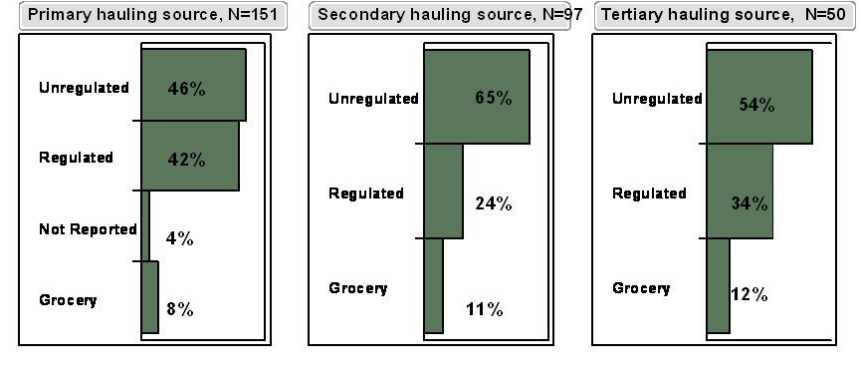
**Percent of participants hauling water from unregulated source, regulated source, and groceries**. Primary, secondary, and tertiary hauling sources are the first three water sources reported by participants in the survey. N = number of participants that use a primary, secondary, and tertiary source.

Water haulers believed that the quality of hauled water was better for their health than regulated and treated NTUA water. In fact, when participants were asked if they thought the NTUA water was good for their health, only 40% responded that it was beneficial. However, when asked if they believed hauled water was good for their health, 60% of participants responded that it was beneficial. Only 15% of respondents reported applying treatment to their hauled water such as filtration or boiling. Interviewees were questioned about the type of water source frequented for hauling. Use of surface water resources such as lakes, stock ponds, and streams are rarely reported, likely due to the ephemeral nature of these sources in this arid environment. Figure 3 summarizes the percentage of respondents hauling water from a regulated water system, grocery store (e.g. bottled water), and unregulated water source (windmill, springs, private well) for the first three water sources reported by each participant in the survey (A, B, & C). On average, participants reported a median travel time of 20 minutes to a water source and haul from at least one source from within their chapter of residence.

#### Environmental contact exposures

Survey participants were asked questions regarding activities that may have influenced exposures through contact with contaminated solids (e.g. soil, sediment, dust, mine materials). They were also questioned about the environmental conditions where they live, including the time spent living in a particular area or participating in certain activities that may have influenced exposure. Survey questions and responses are summarized in Table 2. Because these questions involved participant recall, bias may have influenced their responses. However, the activities reported are consistent with those observed in the field and common to members of these chapters. Because these reported activities provide opportunities for residents to contact environmental conditions that still exist today, responses provide a sound basis for determining likely exposures. In fact, we witnessed children and young adults playing in contaminated sediments and water (Figure 1c). Likewise, contaminated sediment has been disturbed and used for road repairs as well as home construction. New homes as recent as 2005 have been constructed within 300 m of the mine waste pile (Figure 1b).

**Table 2:** Environmental history self-reported by survey participants.

Environmental History	Percent reporting behavior	Median contact years of potential exposure
Lived near a uranium mine: "Near" = downwind, along a road to, in a floodplain of, or within two miles.	40	30

Lived near a uranium mill: "Near" = downwind, along a road to, in a floodplain of, or within two miles.	16	28

Played on a tailings pile or waste dump	20	8

Played outdoors near or next to a uranium mine, mill, or waste dump	18	15

Drinking, wading, or contact with mine water or waste spills	30	5.5

Herding livestock on or next to a uranium mine, mill, or waste dump	25	10

Sheltering livestock in an abandoned mine	4	2

Living in a mining camp	5	7.5

Washing or handling clothes of a friend or family member who was a uranium worker	29	3

Used materials from an abandoned uranium mine or mill for any purpose	28	15

* Not all participants recalled a contact time. Value reported is median of participants who reported a contact time.

Collection of environmental data was necessary to both confirm toxicant exposure in water and contaminated solids and identify areas that present a high risk for present-day exposure based on the behaviors and activities identified through the survey.

### Environmental data collection

#### Water

Sampled water sources were selected by CRUMP and DiNEH staff based upon Navajo Nation Department of Water Resources (NNDWR) databases, local knowledge provided by residents and chapter officials, as well as field reconnaissance [31]. Survey results provided confirmation of which wells were used for human consumption, livestock watering, irrigation, and domestic uses and the amount of water hauled for each purpose. Well selection led to 48 water sources proposed for analysis in the mapping area; however groundtruthing identified 10 of these wells as inoperative, abandoned or inaccessible. The remaining 38 wells represent the most frequented unregulated water resources utilized by participants. CRUMP 2007. reports methods used for water quality sampling and analysis. Water was sampled for potential kidney toxicants (arsenic, cadmium, chromium, copper, mercury, nickel, lead, and uranium), as well as other general chemistry parameters including major ions (calcium, magnesium, potassium, sodium, sulfate, nitrate, carbonate, bicarbonate, fluoride, and chloride) and aesthetic parameters (hardness and total dissolved solids). Results of this preliminary water quality survey indicated that none of the unregulated water resources meet all primary Navajo Nation Safe Drinking Water Act (SDWA) maximum contaminant levels (MCL) or secondary drinking water standards. Drinking water standards adopted by the Navajo Nation Environmental Protection Agency (NNEPA) are identical to those adopted by the U.S. Environmental Protection Agency. Pollutants in excess of primary drinking water standards were (in order of most frequently detected): iron, arsenic, selenium, gross alpha, radium and uranium. Analytes in excess of secondary drinking water standards were sulfate, total dissolved solids, fluoride, and chloride. A discussion of how water quality exceedances result in water-use recommendations for a specific water resource is included in the Map Development and Assumptions section.

#### Sediment

There have been two sediment sampling campaigns through CRUMP and DiNEH to address the spatial distribution of uranium contamination in the mapping area. In total, more than 200 soil/sediment samples were taken between 2004 and 2006 from contaminated and background sites. Surficial and deep (up to 90 cm) samples were taken adjacent to AUM sites, as well as in downstream drainages expected to transport waste during episodic flooding. In addition to the spatial characterization, a detailed geochemical assessment was undertaken to determine the oxidation state, mineral phase, and solubility of uranium minerals present in contaminated samples. These factors govern the mobility and toxicity of uranium. A combination of dissolution experiments and x-ray spectroscopic techniques were utilized to address uranium geochemistry [35]. The results of this study provide critical information to identify high risk areas for mapping. Mean concentrations of uranium downstream of AUM sites were typical of background concentrations of uranium (~3–8 mg/kg) and were comparable to concentrations measured in samples taken in this area in the late 1970's through the National Uranium Resource Evaluation (NURE) [36,37]. However, within ~ 500 m of mine waste pile, concentrations of uranium exceeded 100 mg/kg. Uranium in contaminated material exists as U (VI) in highly soluble mineral forms. When contaminated material is saturated with rain water, pore water concentrations of uranium can exceed 4 mg/L, > 100 times higher than the MCL 30 μg/L. [38]. Such levels present a hazard for use of material or play in standing water and could impact groundwater resources in the area. A further implication for soluble uranium material is that surficial uranium can be easily leached and accumulate deeper in the soil column. Therefore, soil disturbance may result in exposure of unweathered, contaminated material, thereby increasing the risk of exposures to humans and livestock.

### Map development and assumptions

**Figure 4:**
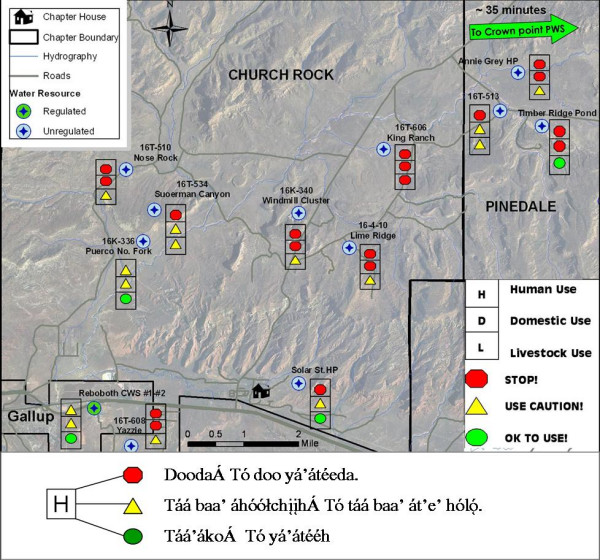
**Water hauling map for Churchrock with use recommendations**. Navajo legend included for human-use recommendations.

**Figure 5:**
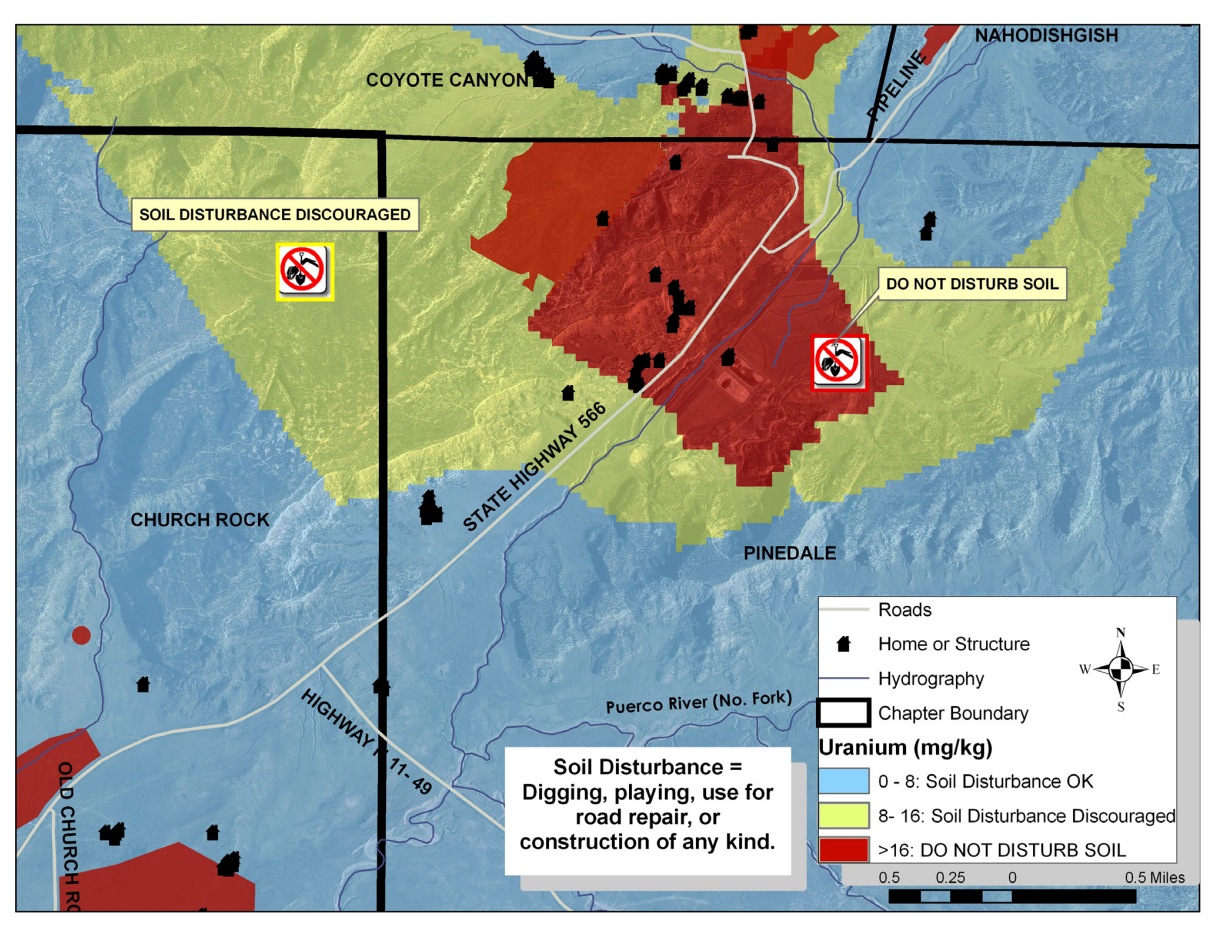
**Soil restriction recommendation map**.

ArcMap 9.2 software (ESRI, Redlands, CA) was used to create maps. Base mapping layers were compiled from the New Mexico Resource Geographic Information Systems Program, McKinley County Geographic Information Systems Center, USEPA region 9, and US Army Corp of Engineers. Key layers used for base map display were 1) 1-m resolution digital RGB orthophotos 2005. for each chapter, 2) chapter boundaries, 3) chapter house locations, 4) roads and driveways, 5) abandoned uranium mine features, and 6) hydrography. Two series of maps were produced: one for water hauling (Figure 4), and one for sediment contamination (Figure 5).

#### Water

A list of water sources was compiled from survey responses, including regulated and unregulated sources being used by participants. Water quality testing resulted in a designation of a water use recommendation for each hauling resource. Water was evaluated for three potential uses: drinking and cooking, livestock watering, and other domestic uses such as cleaning and bathing. Individual analyte results were evaluated for each use by the criteria specified in Table 3. Finally, the overall water quality was reviewed and professional judgment used to ensure the final recommendations considered criteria such as the following:

**Table 3:** Criteria for evaluation of water quality

USE	Comparison Criteria
Drinking or Cooking	NNEPA and USEPA Maximum Contaminant Levels

Livestock Watering	New Mexico Guide M-112, Water Quality for Livestock and Poultry

Other Domestic Use	USEPA Secondary Drinking Water Standards, and overall water chemistry including salinity, TDS, pH, and aesthetic properties

• analytes for which no standards existed,

• sources in which no exceedances of MCLs were observed but more than one toxicant affecting the same target organs were seen at levels within 75% of MCLs,

• situations where TDS or salinity might make water unpalatable.

Use recommendation attributes were symbolized in the software and labeled with both the Navajo Nation Water Source identification number (if applicable), as well as common names used by the community (Figure 4). The symbology for designations is as follows: red pentagon= stop, yellow triangle = use caution, and green circle = ok to use. Approximate travel times to a regulated water source are designated on the map so that if a community member is considering switching to a regulated supply, they will have knowledge of its accessibility. The majority of roads in the mapping area are unpaved and are often impassable during inclement weather; therefore, knowledge the location of alternative safe water sources is critical.

#### Sediment

Sampling coverage included parts of Churchrock, Pinedale, Nahodishgish and Coyote Canyon Chapters where known uranium mining features and mine wastes were present. Where data coverage was missing, current soil uranium concentrations were interpolated from data collected through the NURE program in the late 1970's [37]. The use of NURE data was deemed useful for extrapolation as the concentrations of uranium in the sampling area did not vary significantly from nearby samples taken during the NURE program [35]. However, an exception to this comparison was within an approximate 500 m radius of a mine waste tailings pile where sampled uranium concentrations were >10 times typical uranium soil concentrations [36].

Ordinary kriging analysis was used to create sediment uranium estimation maps using the Geostatistical Analyst tool of ArcGIS 9.2. Where two samples existed at one location (e.g. two different depth), the maximum uranium concentration was used to be more protective of human health. Data were log-transformed and fit using a Gaussian semi-variogram. To designate areas that may contribute to increased exposure to uranium, EPA Region 9 preliminary remediation goals (PRG) were employed to classify the soil uranium estimation maps. PRGs are risk-based concentrations used to evaluate clean-up at contaminated sites and are deemed protective of human health (including sensitive groups) over a lifetime [39]. The PRG addressing the chemical toxicity of uranium is 16 mg/kg based upon direct contact exposures with residential soil [39].

Uranium soil estimation maps were manually classified to define three distinct uranium concentrations and an associated use recommendation over broad geographic areas. The first range, defined in blue color, denoted regions where uranium concentrations fell within reported background concentrations up to 8 mg/kg. The second range, defined in yellow, represented interpolated concentrations exceeding 8 mg/kg. The third range, defined in red, represented interpolated uranium concentrations exceeding the PRG of 16 mg/kg. Any AUM surface features where soil data were not available for interpolation (e.g. sampling not allowed due to private property) were designated in red. Based upon these classifications, the following recommendations were made to minimize exposures through soil disturbance: blue: regular activities okay, yellow: soil disturbance is discouraged, red: no soil disturbance. Soil disturbance can be broadly defined as anything that would create direct contact with the material by stirring it up such as digging in it for play or use in road repair.

Homes and structures (not to scale for confidentiality) were overlaid on the concentration polygons so that community members can associate their residence with a soil disturbance recommendation. A zoom of the mapping area displays these distinct soil exposure areas in Figure 5. It is clear that both grazing and gardening in both the yellow and red areas may result in exposure through food chain contamination. However, because of the cultural reliance on these activities, it is unrealistic to suggest land-use restrictions for grazing and gardening until the community has a spatial understanding of the problem. Imposing restrictions are often inappropriate as they would likely increase financial hardships or create grazing scenarios that are logistically unrealistic. As more data are collected through CRUMP and DiNEH initiatives, Navajo chapter officials will have the required information to pursue clean-up options; a priority to reduce exposures and make decisions with their communities on restricting or relocating grazing, and minimizing recreational activities in these high risk areas. These risk maps are a first step to initiate that exchange of ideas.

Should the communities respond favorably to the risk-mapping strategy, other exposures may be mapped, such as livestock forage. Vegetation samples were taken concomitantly with sediment samples in a limited geographic area to determine if livestock forage had elevated uranium concentrations. Overall, uranium concentrations in grasses ranged from below the detection limit 0.5 mg/kg. to 7.7 mg/kg. Uranium concentrations were not statistically distinguishable between control and contaminated areas. However, mean uranium concentrations were significantly elevated in the roots 5.0 mg/kg. compared to the blade 2.4 mg/kg. These results are comparable to uranium concentrations reported historically, ranging from 0.9 to 3.2 mg/kg in both control and contaminated mining sites in the eastern Navajo chapters [13,14]. It is unclear how the livestock are impacted until tissue analysis is completed on sheep and cattle grazing on contaminated land. However, historical studies do indicate radionuclide uptake in sheep and cattle grazing on uranium contaminated land [13,14]. Paired tissue and forage samples may provide an indication of areas were land use must be managed with care to prevent food chain contamination.

## Results and implications

Figures 4 and 5 provide a detailed view of Churchrock Chapter as an example of the mapping output for both water use recommendations and soil use restrictions, respectively. The Navajo working group (comprised of Navajo project staff and community advisors (n = 10)) that reviewed the first iteration of these maps ranged in age from approximately 30 to 75. Participants were equally male and female and educational background varied from secondary to graduate level. A series of 16 questions were answered and documented so that maps could be revised and displayed in the most productive manner.

### Water use recommendation map

With the exception of some of the elders, all participants that completed this questionnaire were able to understand the use recommendations presented on the map as well as identify the regulated water resource available for use. Additional features were suggested to improve the geographic reference for map readers, including road names and locations of landmarks and stores. When asked if the maps would be clear to a relative or friend who spoke only Navajo, the group was split in their responses. For those who did not believe Navajo speakers would find the map format clear, they also did not believe translating the entire legend into Navajo would improve clarity. This was a significant point, because Navajo is historically only a spoken language and most Navajo do not read or write the language. However, the group consensus was that they would prefer the maps be created in both languages to instill the importance of learning and carrying on the language in younger generations.

The working group was asked questions regarding how they might change their water hauling behaviors after reviewing the maps. With the exception of one person, everyone said that they would be very likely to seek an alternative water source if the map indicated that a hauling source they frequented had more than one red stop light recommendation. However, when asked if they would switch their unregulated hauling source for drinking to a regulated one, the group was also split. Some said they would be very likely to switch while others said they might only consider looking for an alternative. This is not surprising given the high percentage of Navajo that do not believe regulated water is more beneficial to their health. The majority of the working group said they would discontinue domestic water use if it had a red light recommendation, but were split on switching sources if it had a yellow recommendation. The same was true for livestock water use.

### Soil restriction map

Similar to the questions assessing clarity of the water use maps, the working group was able to identify the correct soil restrictions, with the exception of some of the elders. Opinions on clarity for map readers that spoke only Navajo also carried through. Some members of the group thought that no additional features should be added to the map, while others recommended the addition of landmarks and stores as well as the locations of abandoned uranium mines or piles. The majority of the group said they would consider limiting activities (such as riding their hose, walking, or herding sheep) in areas designated in red or in yellow, and unanimously said they would approach a chapter official and request an alternative if they found that their primary designated grazing area had a restricted use recommendation. The group was split when asked if they would consider traveling to an area designated in blue to collect soil to repair a road washout or other use; some responded they would be very likely while others said they would consider it.

### Overall efficacy

All members in this working group had experience communicating about the research project to community members. Based on these past experiences with risk communication in their communities, they were asked how effective these maps would be if kept on display at the chapter houses. On a scale of 1–5, with 1 being ineffective and 5 being very effective, the average response was 4.3, suggesting that the maps are an effective way to disseminate risk information with suggested improvements. Three display options were suggested, including placement in a binder at the chapter house with risk information, in placement in the chapter binder with take-home handouts, and a large format wall display. The group recommended using all three options together as the most effective means of communication. For the permanent placement of maps in the chapter houses, the group felt a written explanation focused on walking the reader through an example would suffice to educate the chapter members on how to use the maps. There was a distinction between the responses between the younger members of the working group compared to the elders. The younger members were more likely to change behavior after seeing these maps, while the older members, albeit not entirely opposed to changing behavior, were more hesitant. This may be a common response for the elders who have had a long history of drinking from particular sources and do not see an obvious relationship with their health problems. Comments from elders over the years often indicate that their concern is for their grandchildren rather than for themselves. The suggestions provided by this working group will be used to make the next iteration of display maps for presentation at chapter houses.

### Limitations

The number and type of maps created are limited by the available data. It is our hope that continuous feedback from community members will highlight which maps are most useful for communicating risk and help to focus field and laboratory data collection efforts in the future. In addition, it is uncertain at this point how the maps will be used by individual chapters and what their overall impact will be. The most significant question is whether our approach produces appropriate and effective responses from community members that would measurably mitigate exposure to toxicants in drinking water and sediment. Future work should focus on incorporating community feedback to produce a more "final" product, and creating a survey instrument to quantify behavioral changes as a result of this method of risk communication. Such iterative response and revision has been a hallmark of the collaboration in this partnership over the nearly decade of working together to understand contamination and health.

### Implications

The use of GIS-based thematic mapping for risk communication provides a novel approach for dissemination of knowledge to impacted communities. Rather than typical risk assessment and communication approaches where restrictions are imposed or unrealistic alternatives are presented, risk-based mapping provides a format that brings the community into the decision-making process. Basic public and environmental health data are easily presented in this visual layout empowering individuals and officials to interact with the information and make decisions regarding daily activities that influence contaminant exposures. Visual renderings of data are preferred over oral presentations of data due to the language barrier, especially among elderly residents who understand little to no English and have limited education in water or soil science. In addition to the accessibility of centrally located maps, the emphasis on these maps being an iterative, rather than a final product is not only empowering to residents, but also increases the relevance of this type of intervention. Since the original preparation of this manuscript, suggestions have been provided to the research team to enhance the maps by developing photos of each of the wells with summaries of contaminants and warnings in English and Navajo. These are being prepared and connected to the wall maps as well as inserted in the chapter binders to ensure the project remains responsive to the needs of the communities, and provides information in diverse ways the help to meet the needs of community members and bridge the differences between cultures.

As researchers continue to do work in Native American communities, it is our hope that they begin to incorporate non-traditional approaches to risk communication such as the method presented here. Strategies such as this are educational and empowering to residents. More importantly, they provide a means to inform decisions made *by *the impacted communities instead of *for *them by outsiders. Strategies such as the mapping described here can provide a public health intervention through stimulating behavior change through informed decision-making. The comprehensive nature of information provided reduces frustration and fear by providing sufficient information to prioritize activities and take action at an individual level. The work described here is not intended to drive remedial action, although these tools can be used by communities to support their requests to policy- and decision-makers, furthering the empowerment of the residents.

## Abbreviations

GIS: Geographic Information Systems; DiNEH: Diné Network for Environmental Health; ENHB: Eastern Navajo Health Board; CRUMP: Churchrock Uranium Monitoring Project; NTUA: Navajo Tribal Utility Authority; NAAQS: National Ambient Air Quality Standard; EPA: Environmental Protection Agency; NOAA: National Oceanographic and Atmospheric Administration; NNDWR: Navajo Nation Department of Water Resources; SDWA: Safe Drinking Water Act; NNEPA: Navajo Nation Environmental Protection Agency; MCL: Maximum Contaminant Level; PRG: preliminary remediation goals; EPA: Environmental Protection Agency; TDS: Total dissolved solids; CEHW: Community Environmental Health Worker.

## Competing interests

The authors declare that they have no competing interests.

## Authors' contributions

Jd conceived of and designed the mapping strategy and prepared the manuscript. DB provided expertise on risk communication in Native communities and assisted in manuscript preparation. MC and MD provided survey data and helped organize and collect information from the working group. JD provided GIS support and assisted in manuscript preparation. CG, TR, and TN assisted in environmental and geospatial data collection used to create maps. SHA, TM, BS collected health survey data used in this manuscript and provided Navajo translation. CS provided CRUMP data and assisted in manuscript preparation. JL is the principal investigator for the DiNEH project and provided assistance in developing the mapping and communication strategy as well organizing the working group and in manuscript preparation. All authors read and approved the final manuscript.
